# Supplementary material for: Palmitic Acid Impairs Myogenesis and Alters Temporal Expression of miR-133a and miR-206 in C2C12 Myoblasts
Source: Int J Mol Sci. 2021 Mar 9;22(5):2748. doi: 10.3390/ijms22052748 (PMC7963199; doi:10.3390/ijms22052748)
Supplement: Supplementary file 1 [file ijms-22-02748-s001.pdf]

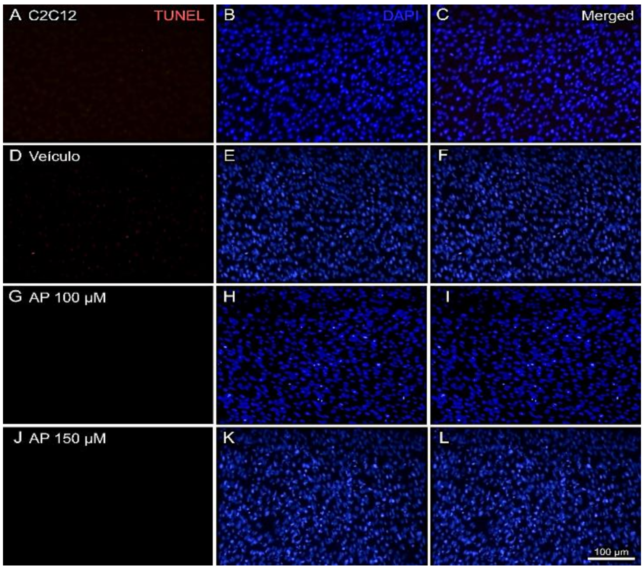

Supplementary Figure S1. PA does not induce apoptosis in C2C12 cells. TUNEL assay: myoblasts were immunolabeled with tetramethyl rhodamine (red) or DAPI (blue), $n = 3$.
